# Genetic activation of Nrf2 reduces cutaneous symptoms in a murine model of Netherton syndrome

**DOI:** 10.1242/dmm.042648

**Published:** 2020-06-01

**Authors:** Sukalp Muzumdar, Michael Koch, Hayley Hiebert, Andreas Bapst, Alessia Gravina, Wilhelm Bloch, Hans-Dietmar Beer, Sabine Werner, Matthias Schäfer

**Affiliations:** 1 Department of Biology, Institute of Molecular Health Sciences, ETH Zurich, 8093 Zurich, Switzerland; 2 Department of Molecular and Cellular Sport Medicine, German Sport University Cologne, 50933 Cologne, Germany; 3 Department of Dermatology, University Hospital of Zurich, Gloriastrasse 3, 8091 Zurich, Switzerland

**Keywords:** Epidermal barrier, Inflammation, Netherton syndrome, Nrf2

## Abstract

Netherton syndrome is a monogenic autosomal recessive disorder primarily characterized by the detachment of the uppermost layer of the epidermis, the *stratum corneum*. It results from mutations in the *SPINK5* gene, which codes for a kallikrein inhibitor. Uncontrolled kallikrein activity leads to premature desquamation, resulting in a severe epidermal barrier defect and subsequent life-threatening systemic infections and chronic cutaneous inflammation. Here, we show that genetic activation of the transcription factor nuclear factor (erythroid-derived 2)-like 2 (Nfe2l2/Nrf2) in keratinocytes of *Spink5* knockout mice, a model for Netherton syndrome, significantly alleviates their cutaneous phenotype. Nrf2 activation promoted attachment of the *stratum corneum* and concomitant epidermal barrier function, and reduced the expression of pro-inflammatory cytokines such as tumor necrosis factor α and thymic stromal lymphopoietin. Mechanistically, we show that Nrf2 activation induces overexpression of secretory leukocyte protease inhibitor (Slpi), a known inhibitor of kallikrein 7 and elastase 2, in mouse and human keratinocytes *in vivo* and *in vitro*, respectively. In the Spink5-deficient epidermis, the upregulation of Slpi is likely to promote stabilization of corneodesmosomes, thereby preventing premature desquamation. Our results suggest pharmacological NRF2 activation as a promising treatment modality for Netherton syndrome patients.

This article has an associated First Person interview with the first author of the paper.

## Abstract (precis)

**Summary:** Nrf2 activation ameliorates the epidermal barrier defect and cutaneous inflammation in a mouse model of Netherton syndrome, suggesting the utility of NRF2-activating compounds in patients with this genetic disease.

## INTRODUCTION

Netherton syndrome (also known as Comèl-Netherton syndrome) is a severe, monogenic, autosomal recessive disorder that is clinically characterized by the triad of congenital redness and scaling of the skin (erythroderma), the development of erythematous plaques with double-edged scales (*ichthyosis linearis circumflexa*) and hair shaft abnormalities (*trichorrhexis invaginata*) (Comèl, 1949; Netherton, 1958). Netherton syndrome is caused by mutational inactivation of the serine protease inhibitor Kazal-type 5 (*SPINK5*) gene, which encodes lympho-epithelial Kazal-type-related inhibitor (LEKTI) (Chavanas et al., 2000a,b). LEKTI is responsible for the inhibition of two kallikrein proteases (KLK5 and KLK7). Both these kallikreins are essential for the desquamation of the uppermost layer of the skin, the *stratum corneum*, through the cleavage of corneodesmosomal proteins such as corneodesmosin (CDSN) and desmoglein 1 (DSG1) (Caubet et al., 2004; Deraison et al., 2007; Descargues et al., 2005; Yang et al., 2004). The inactivation of LEKTI in Netherton syndrome leads to the unchecked activity of these proteases, resulting in enhanced corneocyte desquamation and a concomitant epidermal permeability barrier defect (Komatsu et al., 2002). This defect increases transepidermal water loss and also enables the penetration of pathogens, allergens and irritants into the skin. As a consequence, patients suffer from generalized erythroderma and atopy and are frequently affected by infections (Judge et al., 1994). LEKTI is also known to inhibit KLK14, which together with KLK5 directly drives the inflammatory and atopic symptoms associated with this disease (Briot et al., 2009). Furthermore, owing to the unchecked activity of KLK5, the zymogen form of elastase 2 (ELA2; also known as ELANE) is cleaved and hyperactivated, further exacerbating the barrier dysfunction through the misprocessing of (pro)-filaggrin and of lipids in the *stratum corneum* (Bonnart et al., 2010). Complications arising from this barrier defect can be lethal early in life, especially through infections and/or severe (neonatal) hypernatremic dehydration (Giroux et al., 1993; Jones et al., 1986; Stoll et al., 2001).

To study the molecular mechanisms underlying Netherton syndrome, mouse models have been developed either by deletion of the entire *Spink5* locus (Yang et al., 2004), by deletion of the 5′ end of the gene locus (Descargues et al., 2005) or by targeted gene disruption, mimicking mutations described in patients (Hewett et al., 2005). In all of these models, except for a recently described mosaic model (Kasparek et al., 2016), homozygous mutants exhibit perinatal lethality owing to dehydration caused by the dysfunctional epidermal permeability barrier, although variations in their phenotypic abnormalities have been described. The mouse model used in this study was shown to exhibit a premature proteolytic breakdown of Cdsn, which probably makes a major contribution to the barrier dysfunction, whereas Dsg1 and desmocollin 1 (Dsc1) expression were unaffected (Yang et al., 2004).

Nuclear factor (erythroid-derived 2)-like 2 (Nfe2l2, Nrf2) is a master regulator of the cellular antioxidant defense system. It is activated in the presence of oxidative or electrophilic stressors, which stabilize Nrf2. Newly formed Nrf2 then translocates to the nucleus and regulates the transcription of its downstream targets (Sykiotis and Bohmann, 2010). These targets include genes coding for various antioxidant proteins, phase II detoxification enzymes and drug transporters, resulting in a global cytoprotective response (Yamamoto et al., 2018). A large number of additional Nrf2 target genes have been described; for example, genes involved in the unfolded protein response, in the formation of atherosclerotic plaques, in purine biosynthesis (Harada et al., 2012; Meakin et al., 2014; Mitsuishi et al., 2012; Rolfs et al., 2015; Tonelli et al., 2018) and in extracellular matrix production (Hiebert et al., 2018).

In the skin, Nrf2 has been implicated in the protection of keratinocytes from the toxicity of xenobiotics, such as arsenite, cumene hydroperoxide and sulfur mustard analogs (Schäfer and Werner, 2015). Furthermore, pharmacological activation of Nrf2 protected keratinocytes from UVB-induced apoptosis and reduced UV- and mutagen-induced skin tumorigenesis in mice (Dinkova-Kostova et al., 2006; Kleszczyński et al., 2013; Xu et al., 2006). However, Nrf2 activation can also lead to pro-tumorigenic metabolic changes in the early stages of carcinogenesis, thereby promoting skin tumorigenesis in mutagen-independent murine skin cancer models (Rolfs et al., 2015).

We previously generated mice that express a well-characterized constitutively active mutant of Nrf2 (caNrf2) under the control of a cytomegalovirus (CMV) enhancer and a β-actin promoter (CMV-caNrf2 mice) in the presence of Cre recombinase. Mice expressing Cre under the control of the keratin 5 (K5) promoter (Ramirez et al., 2004) were used to direct and restrict caNrf2 expression to keratinocytes. Importantly, the level of Nrf2 target gene activation seen in these mice is comparable with the level achieved upon pharmacological activation of Nrf2 in mouse skin *in vivo* and in mouse keratinocytes *in vitro* (Schäfer et al., 2012). Therefore, the transgene mimics the effect seen with pharmacological Nrf2 activators. The K5cre-CMVcaNrf2 transgenic mice exhibited acanthosis, hyperkeratosis and mild, chronic cutaneous inflammation starting at around postnatal day 10 (Schäfer et al., 2012, 2014). The hyperkeratosis resulted, at least in part, from Nrf2-mediated upregulation of secretory leukocyte protease inhibitor (Slpi) (Schäfer et al., 2014), an inhibitor of the proteases Klk7 and Ela2 (Bonnart et al., 2010; Franzke et al., 1996; Schäfer et al., 2014). As Nrf2 activation had previously been shown to stabilize the impaired epidermal barrier of loricrin knockout mice during embryonic development (Huebner et al., 2012), we hypothesized that activating Nrf2 could bring about beneficial effects in the context of *Spink5* deficiency. Therefore, we studied the consequences of genetic Nrf2 activation on the phenotype of *Spink5* knockout mice and attempted to unravel the underlying molecular mechanisms.

## RESULTS

### Genetic activation of Nrf2 in *Spink5* knockout mice

**Fig. 1.**
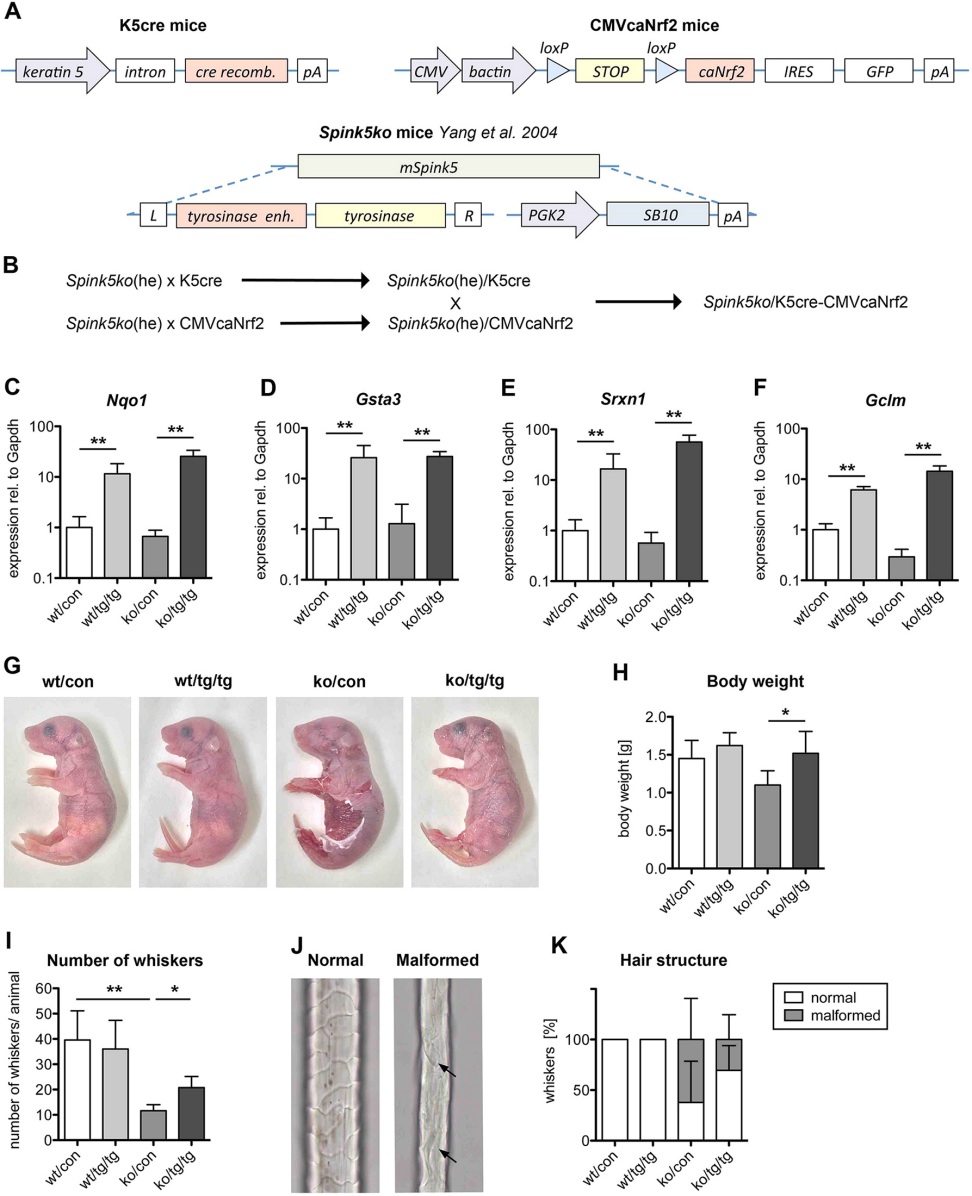
**Genetic activation of Nrf2 partially rescues the skin phenotype of Spink5ko mice.** (A) Scheme of the constructs used to generate K5cre mice (upper left), CMVcaNrf2 mice (upper right) and Spink5ko mice (lower scheme). (B) Generation of Spink5ko/K5cre-CMVcaNrf2 mice by crossing progeny of Spink5ko/K5cre and Spink5ko/CMVca-Nrf2 mice. (C**-**F) qRT-PCR for *Nqo1* (C), *Gsta3* (D), *Srxn1* (E) and *Gclm* (F) relative to *Gapdh* using RNA isolated from the epidermis of control (wt/con), K5cre-CMVcaNrf2 (wt/tg/tg), Spink5ko (ko/con) and Spink5ko/K5cre-CMVcaNrf2 (ko/tg/tg) mice; *n*=5-7 mice per genotype. (G) Lateral view of representative newborn wt/con, wt/tg/tg, ko/con and ko/tg/tg mice. Note the widespread *stratum corneum* detachment in ko/con mice, but almost normal *stratum corneum* attachment in ko/tg/tg mice; *n*≥17 mice per genotype. (H) Weight of newborn wt/con, wt/tg/tg, ko/con and ko/tg/tg mice; *n*=3-22 mice per genotype. (I) Number of whiskers in wt/con, wt/tg/tg, ko/con and ko/tg/tg newborn mice; *n*=4-5 mice per genotype. (J,K) Representative pictures of normal and malformed whiskers from wt/con and ko/con mice (J) and number of normal and malformed whiskers in wt/con, wt/tg/tg, ko/con and ko/tg/tg newborn mice (K); *n*=4 mice per genotype. All bar graphs represent mean±s.d. *P≤0.05, **P≤0.01, Mann–Whitney *U*-test. he, heterozygous; Spink5ko always denotes homozygous.

To assess the consequences of Nrf2 activation in a murine model of Netherton syndrome, *Spink5* knockout mice (Spink5ko) (Yang et al., 2004) and transgenic mice expressing caNrf2 in keratinocytes (K5cre-CMVcaNrf2) (Schäfer et al., 2012) were used for the generation of mice lacking *Spink5* and expressing caNrf2 in keratinocytes using a three-step breeding scheme (Fig. 1A,B). The resulting Spink5ko/K5cre-CMVcaNrf2 mice (designated ko/tg/tg – homozygous knockout for *Spink5* and hemizygous for both *K5-Cre* and *caNrf2* transgenes) constitutively express the caNrf2 mutant in all keratinocytes of the epidermis and pilosebaceous unit, owing to the activity of the K5 promoter in basal keratinocytes of the epidermis and the outer root sheath of hair follicles (Ramirez et al., 2004). The deletion of the transcription/translation STOP cassette in these cells results in transgene expression in all keratinocytes of the epidermis and the pilosebaceous unit (Fig. 1A,B). K5cre (wt/tg/wt) mice, CMVcaNrf2 (wt/wt/tg) mice without Cre and wild-type mice (wt/wt/wt) were used as controls. All mice were sacrificed and analyzed within the first 12 h after birth to avoid death from desiccation.

The appropriate expression of the caNrf2 transgene has been demonstrated earlier (Schäfer et al., 2012). Quantitative reverse-transcription PCR (qRT-PCR) analysis of epidermal RNA from Spink5ko/K5cre-CMVcaNrf2 mice revealed a strong upregulation of the classical Nrf2 transcriptional target genes NAD(P)H dehydrogenase (quinone) 1 (*Nqo1*), glutathione *S*-transferase A3 (*Gsta3*), sulfiredoxin 1 (*Srxn1*) and glutamate-cysteine ligase, modifier subunit (*Gclm*) compared with control mice (Fig. 1C-F) (Chorley et al., 2012; McWalter et al., 2004). Activation of these classical Nrf2 target genes was not observed in Spink5ko compared with control mice, suggesting a lack of compensatory Nrf2 activation in the keratinocytes of these mice upon loss of a functional *Spink5* gene (Fig. 1C-F).

### Normalization of the macroscopic appearance of Spink5ko mice by activation of Nrf2

At birth, Spink5ko (ko/con) mice exhibit a strongly reduced attachment of the *stratum corneum* all over the body, a hallmark of Netherton syndrome (Fig. 1G) (Hausser and Anton-Lamprecht, 1996). This phenotype was even more pronounced than in other murine models of Netherton syndrome with a different mutation and in the C57BL/6 strain (Descargues et al., 2005). Attachment of the *stratum corneum* was largely restored in Spink5ko/K5cre-CMVcaNrf2 (ko/tg/tg) pups, although the extremities were often still affected. This might be the result of incomplete recombination in the newborn mice, resulting in patchy expression of the *caNrf2* transgene. K5cre-CMVcaNrf2 (wt/tg/tg) mice did not exhibit detachment of the *stratum corneum* and were macroscopically indistinguishable from control (wt/con) mice at birth (Fig. 1G), as the hyperkeratosis in these mice only develops at around postnatal day 10 (Schäfer et al., 2012). The reduced body weight of Spink5ko mice observed after birth was rescued in Spink5ko/K5cre-CMVcaNrf2 mice (Fig. 1H).

Although Spink5ko pups do not display the pathognomic *trichorrhexis invaginata* due to the early stage in hair development, they had fewer whiskers compared with control and K5cre-CMVcaNrf2 pups and the remnant whiskers were structurally abnormal, thinner and fragile (Fig. 1I-K). Spink5ko/K5cre-CMVcaNrf2 pups displayed a significant increase in the number of whiskers compared with Spink5ko mice and exhibited a higher proportion of normal whiskers (Fig. 1I-K).

### Normalization of *stratum corneum* integrity and keratinocyte differentiation in Spink5ko/K5cre-CMVcaNrf2 mice

**Fig. 2.**
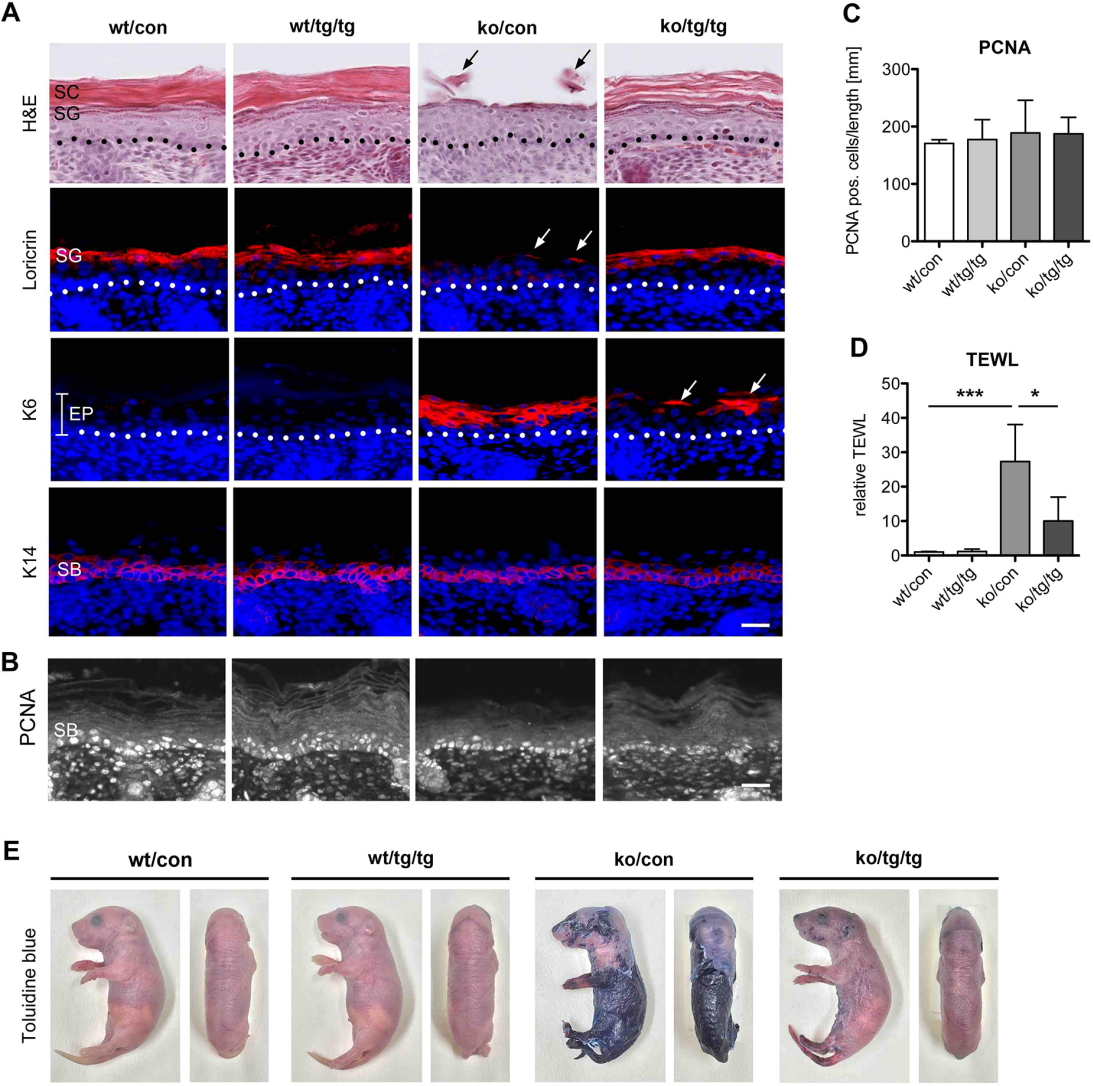
**Nrf2 activation partially rescues epidermal differentiation and barrier function abnormalities in Spink5ko mice.** (A) Representative photomicrographs showing H&E staining (top row) or immunofluorescence staining for loricrin (second row), K6 (third row) and K14 (bottom row) (all in red) and counterstaining of nuclei with Hoechst (blue) on transverse back skin sections of wt/con, wt/tg/tg, ko/con and ko/tg/tg mice. Dotted lines indicate the basement membrane. The arrows point to remnant *stratum corneum* in the top row, loricrin-positive cells in the second row and K6-positive cells in the third row. (B,C) PCNA staining (B) and quantification of PCNA-positive cells in the epidermis (C) of wt/con, wt/tg/tg, ko/con and ko/tg/tg mice; *n*=3-4 mice per genotype. (D) Transepidermal water loss (TEWL) in wt/con, wt/tg/tg, ko/con and ko/tg/tg newborn mice; *n*=6-21 mice per genotype. (E) Lateral (left) and dorsal (right) view of Toluidine Blue-treated wt/con, wt/tg/tg, ko/con and ko/tg/tg newborn mice. All bar graphs represent mean±s.d. *P≤0.05, ***P≤0.001, Mann–Whitney *U*-test. Scale bars: 25 μm. EP, epidermis; SB, *stratum basale*; SG, *stratum granulosum*.

Histological analysis of the skin of Spink5ko (ko/con) newborn mice revealed an almost complete loss of the *stratum corneum*, resulting from the loss of this poorly attached cell layer either *in vivo* or during processing for paraffin embedding, sectioning or staining/immunolabeling (Fig. 2A, top row). Histological analysis further confirmed the loss of the granular layer, as evidenced by the almost complete lack of expression of the late differentiation marker loricrin compared with wt/con and wt/tg/tg mice (Fig. 2A, second row). As previously described, and indicative of a disturbed epidermal differentiation program, Spink5ko mice also exhibited interfollicular expression of keratin 6 (K6; also known as Krt6), in contrast to its typical follicular expression pattern in control mice (Fig. 2A, third row) (Yang et al., 2004). Keratin 14 (K14) expression was appropriately restricted to the basal layer in Spink5ko mice (Fig. 2A, bottom row), as described earlier for this specific mouse model (Yang et al., 2004).

By contrast, Spink5ko/K5cre-CMVcaNrf2 (ko/tg/tg) mice exhibited a better attachment of the *stratum corneum* and the *stratum granulosum*, as reflected by the normal expression of loricrin. Furthermore, interfollicular expression of K6 was restricted to only a few patches, indicating a partial restoration of epidermal differentiation (Fig. 2A).

Expression of proliferating cell nuclear antigen (PCNA) was normal in Spink5ko mice, indicating a lack of compensatory keratinocyte proliferation at this stage (Fig. 2B,C). This observation is consistent with results from 5-bromo-2′-deoxyuridine (BrdU) incorporation assays previously performed in the same mouse model (Yang et al., 2004).

Overall, these analyses demonstrate that genetic Nrf2 activation restored the *stratum corneum* integrity and also partially ameliorated the disturbed epidermal differentiation program in Spink5ko mice.

### Restoration of epidermal barrier function in Spink5ko/K5cre-CMVcaNrf2 mice

Epidermal barrier functionality was evaluated by transepidermal water loss (TEWL, a quantitative read-out for the inside-out barrier; Indra and Leid, 2011) and by Toluidine Blue penetration assay (a read-out for the outside-in barrier; Schmitz et al., 2015). TEWL was increased 27-fold in neonatal Spink5ko (ko/con) versus control (wt/con) mice. This abnormality was partially rescued in Spink5ko/K5cre-CMVcaNrf2 (ko/tg/tg) mice: TEWL was reduced by more than 60% compared with Spink5ko mice (Fig. 2D). Furthermore, although almost the entire body surface of Spink5ko mice was Toluidine Blue permeable, the stain was only observed in some patches of the extremities of Spink5ko/K5cre-CMVcaNrf2 mice (Fig. 2E). Neither control nor K5cre-CMVcaNrf2 mice showed Toluidine Blue uptake (Fig. 2E). Thus, genetic Nrf2 activation led to a significant, although partial, regression of the barrier defect present in newborn Spink5ko mice.

### Nrf2 activation reduces expression of pro-inflammatory cytokines and chemokines in Spink5ko mice

**Fig. 3.**
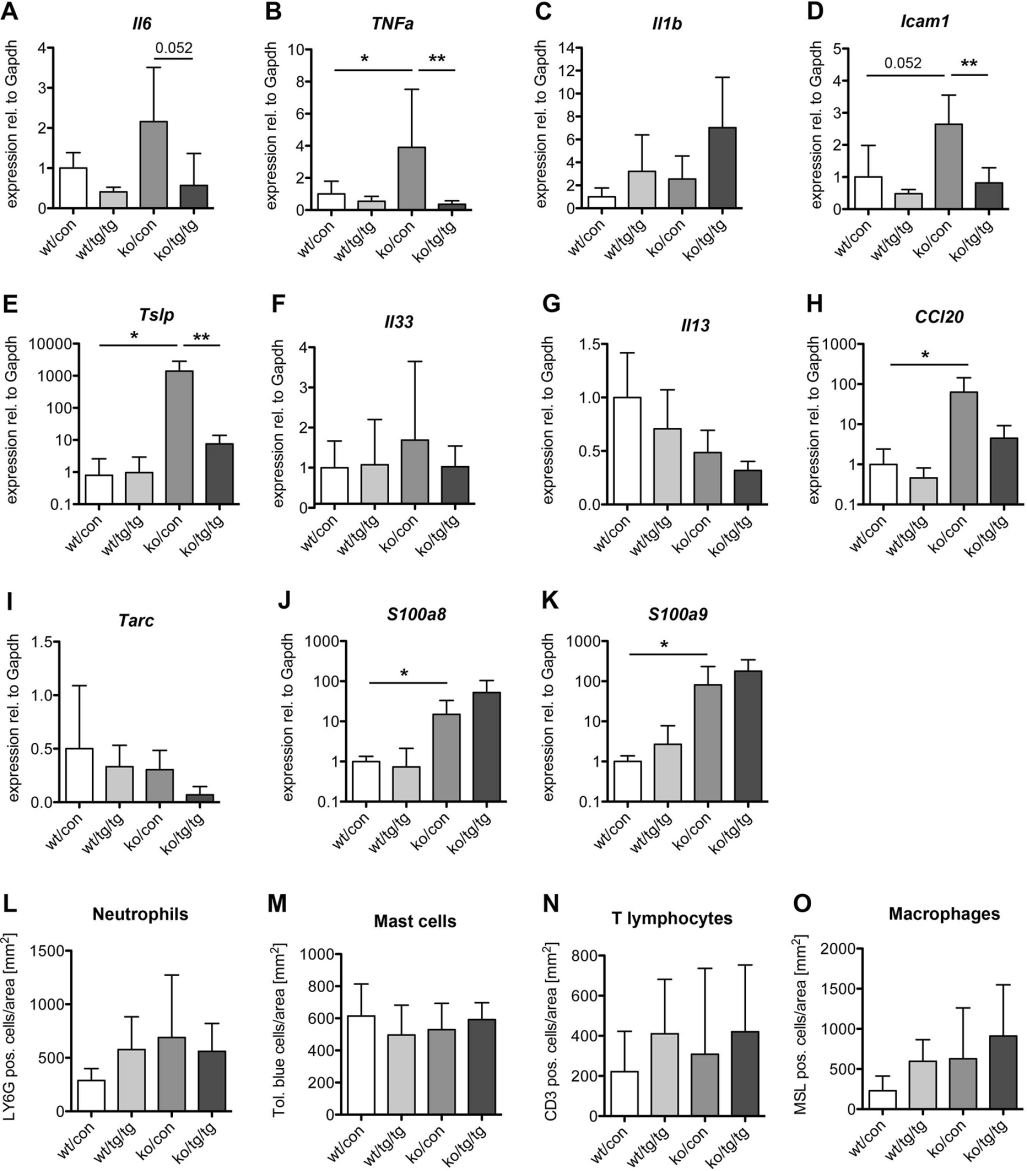
**Nrf2 activation partially rescues skin inflammation in Spink5ko mice.** (A-K) qRT-PCR for *Il6* (A), *Tnfa* (B), *Il1b* (C), *Icam1* (D), *Tslp* (E), *Il33* (F), *Il13* (G), *Ccl20* (H), *Tarc* (I), *S100a8* (J) and *S100a9* (K) relative to *Gapdh* using RNA from the epidermis of wt/con, wt/tg/tg, ko/con and ko/tg/tg newborn mice; *n*=4-8 mice per genotype. (L-O) Number of Ly6G-positive neutrophils (immunofluorescence staining) (L), Toluidine Blue-stained mast cells (M), CD3-positive T-lymphocytes (immunofluorescence staining) (N) and MSL-positive macrophages (immunofluorescence staining) (O) per area of the dermis in wt/con, wt/tg/tg, ko/con and ko/tg/tg newborn mice. All bars represent mean±s.d.; *n*=3-6 mice per genotype. *P≤0.05, **P≤0.01, Mann–Whitney *U*-test.

It has been previously described that Spink5ko mice overexpress several pro-inflammatory cytokines and chemokines as a consequence of the barrier defect (Descargues et al., 2005; Hewett et al., 2005). qRT-PCR analysis of epidermal RNA indeed revealed a significantly increased expression of the gene encoding tumor necrosis factor α (*Tnfa*; also known as *Tnf*) and a mild increase in interleukin-6 (*Il6*) expression in Spink5ko mice (Fig. 3A,B). Expression of these genes was normalized in Spink5ko/K5cre-CMVcaNrf2 mice (Fig. 3A,B).

By contrast, expression of interleukin-1β (*Il1b*) was not significantly changed at this early stage in the Spink5ko mouse line used in this study, and was even slightly upregulated in Spink5ko/K5cre-CMVcaNrf2 pups (Fig. 3C).

The inflammatory reaction in Spink5ko mice is driven at least in part by the protease-activated receptor 2 (Par2)–nuclear factor κB (NF-κB) axis (Briot et al., 2010). Indeed, the classical downstream targets of this signaling axis, intercellular adhesion molecule 1 (*Icam1*) and thymic stromal lymphopoietin (*Tslp*) (Buddenkotte et al., 2005; Kouzaki et al., 2009; Moniaga et al., 2013), were upregulated in Spink5ko mice, and this was partially or even fully rescued by caNrf2 expression (Fig. 3D,E). The Th2-associated alarmin interleukin-33 (*Il33*) and the Th2-promoting interleukin-13 (*Il13*) were not upregulated in Spink5ko mice and were also unchanged in Spink5ko/K5cre-CMVcaNrf2 mice (Fig. 3F,G). Furthermore, although the expression of the dendritic cell-attracting chemokine ‘(C-C motif) ligand 20’ (*Ccl20*) was upregulated in Spink5ko mice and partially normalized in Spink5ko/K5cre-CMVcaNrf2 mice, the expression of the T-cell attractant *Tarc* (also known as *Ccl17*) was unaffected upon *Spink5* deletion (Fig. 3H,I). Finally, an increased expression of the genes encoding the antimicrobial peptides/chemokines S100a8 and S100a9, an early sign of keratinocyte stress/injury that precedes an influx of inflammatory cells (Voss et al., 2012), was observed in Spink5ko pups, but this was not rescued by caNrf2 expression (Fig. 3J,K).

Despite the upregulation of some pro-inflammatory cytokines in the skin of newborn Spink5ko mice, there was no increase in the numbers of lymphocyte antigen 6 complex locus G6D (Ly6G)-positive neutrophils, Toluidine Blue-stained mast cells, CD3-positive T-lymphocytes or macrophage-specific lectin (MSL)-positive macrophages (Fig. 3L-O). Numbers of these immune cells were also unaffected by expression of the *caNrf2* transgene at this early time point (Fig. 3L-O).

### Nrf2 activation promotes cell-cell adhesion in Spink5ko mice

**Fig. 4.**
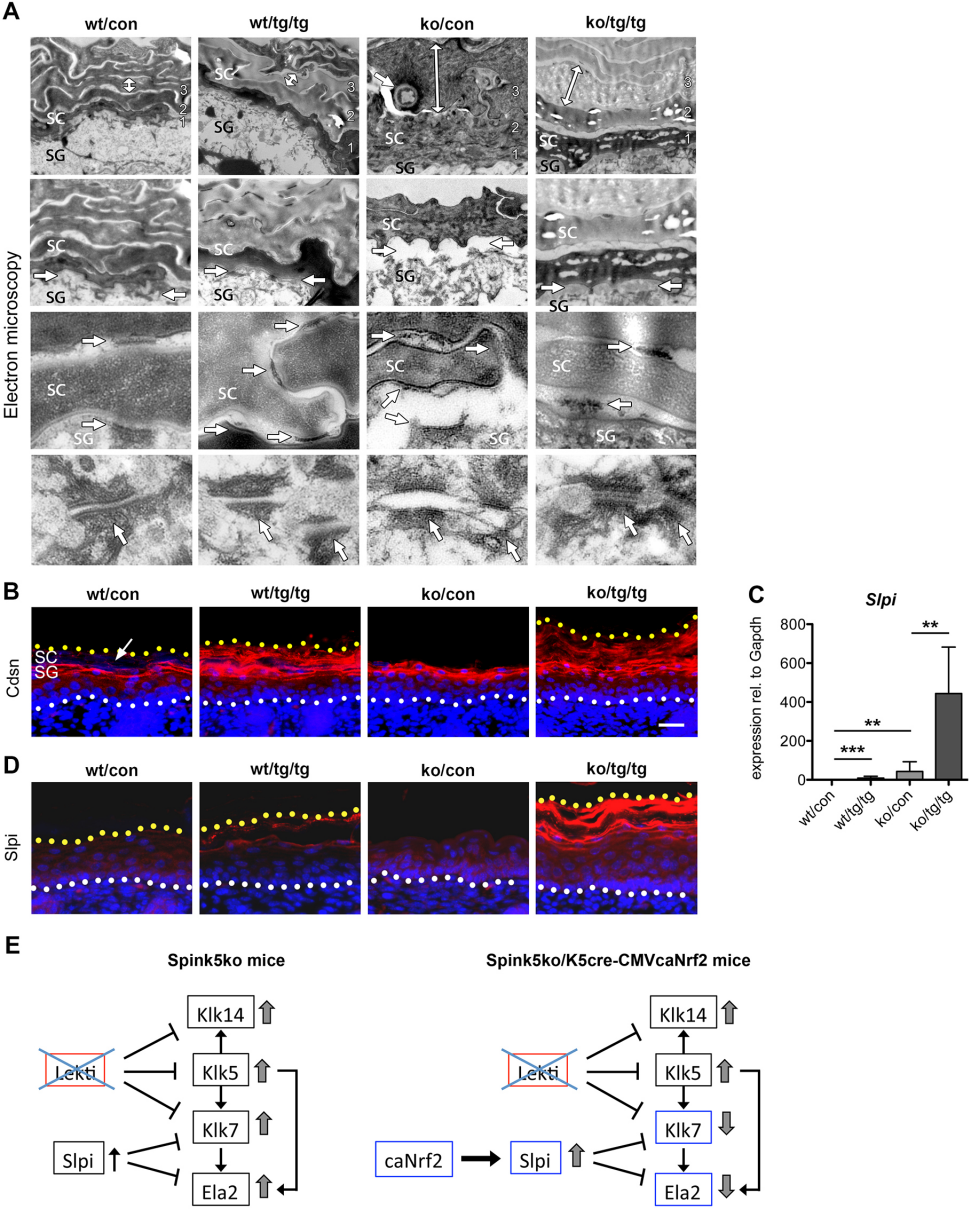
**Nrf2 activation promotes cell-cell adhesion in the epidermis of Spink5ko mice.** (A) Electron microscopy images of the *stratum corneum* (top, second and third rows) and *stratum granulosum* (bottom row) in wt/con, wt/tg/tg, ko/con and ko/tg/tg newborn mice. Double-headed arrows in the top row indicate the thickness of corneocytes. Arrows in the second row demarcate the attachment of the *stratum corneum* to the *stratum granulosum*. Arrows in the third and bottom rows point to (corneo)desmosomes in the *stratum corneum* and *stratum granulosum*, respectively. (B) Immunofluorescence staining of corneodesmosin (Cdsn) (red) and counterstaining of nuclei with Hoechst (blue) on transverse back skin sections of wt/con, wt/tg/tg, ko/con and ko/tg/tg newborn mice. The white dotted line marks the position of the basement membrane and the yellow dotted line the upper edge of the *stratum corneum*. The arrow points to the Cdsn-negative upper *stratum corneum* of wt/con mice. (C) qRT-PCR for *Slpi* relative to *Gapdh* using RNA from the epidermis of wt/con, wt/tg/tg, ko/con and ko/tg/tg newborn mice; *n*=5-9 mice per genotype. (D) Immunofluorescence staining for Slpi on transverse back skin paraffin sections of wt/con, wt/tg/tg, ko/con and ko/tg/tg newborn mice. Note the strong Slpi staining in the granular layer of ko/tg/tg mice. (E) Working model. Left: in the epidermis of Spink5ko mice, the loss of Lekti leads to unchecked activation of Klk5, Klk7, Klk14 and Ela2. Slpi expression is slightly upregulated; however, it is not sufficient to compensate for the lack of Lekti. Right: in Spink5ko/K5cre-CMVcaNrf2 mice, caNrf2 partially compensates for the lack of Lekti by Slpi-mediated inhibition of Klk7 and Ela2. All bars represent mean±s.d. **P≤0.01, ***P≤0.001, Mann–Whitney *U*-test. Scale bars: 25 μm. SC, *stratum corneum*; SG, *stratum granulosum*.

To determine the effect of caNrf2 expression on cell-cell adhesion in Spink5ko mice, we performed ultrastructural analysis of the epidermis. Spink5ko (ko/con) mice exhibited hyperplasia of the upper corneocytes (Fig. 4A, top row), a detachment of the cornified layers (Fig. 4A, second row) and a rupture of the corneodesmosomes in the *stratum corneum* (Fig. 4A, third row) and of the desmosomes in the *stratum granulosum* (Fig. 4A, bottom row) compared with control (wt/con) or K5cre-CMVcaNrf2 (wt/tg/tg) mice.

By contrast, Spink5ko/K5cre-CMVcaNrf2 (ko/tg/tg) mice exhibited thinner corneocytes, a better attachment of the cornified layers and more intact (corneo)desmosomes in the *stratum corneum* and *stratum granulosum* when compared with Spink5ko mice (Fig. 4A). These findings indicate that Nrf2 activation enhanced the attachment of the *stratum corneum* by promoting (corneo)desmosome-mediated cell-cell adhesion in the epidermis.

Corneodesmosin is a structural protein present in desmosomes and corneodesmosomes that is cleaved by kallikreins in the *stratum corneum* (Caubet et al., 2004). Interestingly, several layers of corneodesmosin-positive cells were detected in K5cre-CMVcaNrf2 and Spink5ko/K5cre-CMVcaNrf2 mice, extending to the upper *stratum corneum*. By contrast, corneodesmosin staining was restricted to the upper viable epidermal layers and lower *stratum corneum* in control mice and to one to two layers of differentiated (but viable) cells in Spink5ko mice (Fig. 4B). The broad corneodesmosin staining in mice expressing the *caNrf2* transgene probably reflects the protection from protease-mediated cleavage, providing a possible explanation for the restored attachment of the *stratum corneum* and the normalization of the structure of desmosomes and corneodesmosomes in Spink5ko/K5cre-CMVcaNrf2 mice.

It was previously shown that K5cre-CMVcaNrf2 mice overexpress Slpi, a direct transcriptional target of Nrf2 in the epidermis (Schäfer et al., 2012). qRT-PCR using RNA from the entire epidermis revealed a ninefold increase in *Slpi* expression in K5cre-CMVcaNrf2 (wt/tg/tg) versus control (wt/con) mice (Fig. 4C). Interestingly, a 43-fold *Slpi* upregulation was observed in Spink5ko (ko/con) mice (Fig. 4C). This increase is probably a consequence of Nrf2-independent mechanisms, as other classical Nrf2 targets were not upregulated in Spink5ko (ko/con) mice (see Fig. 1C-F). In Spink5ko/K5cre-CMVcaNrf2 mice, *Slpi* expression was upregulated 443-fold compared with control mice and 10-fold upregulated compared with Spink5ko mice (Fig. 4C). Immunofluorescence staining revealed a mild increase in Slpi expression in K5cre-CMVcaNrf2 mice and a very strong increase in Spink5ko/K5cre-CMVcaNrf2 mice, particularly in the *stratum granulosum* and *stratum corneum* (Fig. 4D). Owing to the detachment of these layers in Spink5ko pups (Fig. 2A, top and second rows, Fig. 4D), the levels of Slpi protein could not be determined in these pups.

**Fig. 5.**
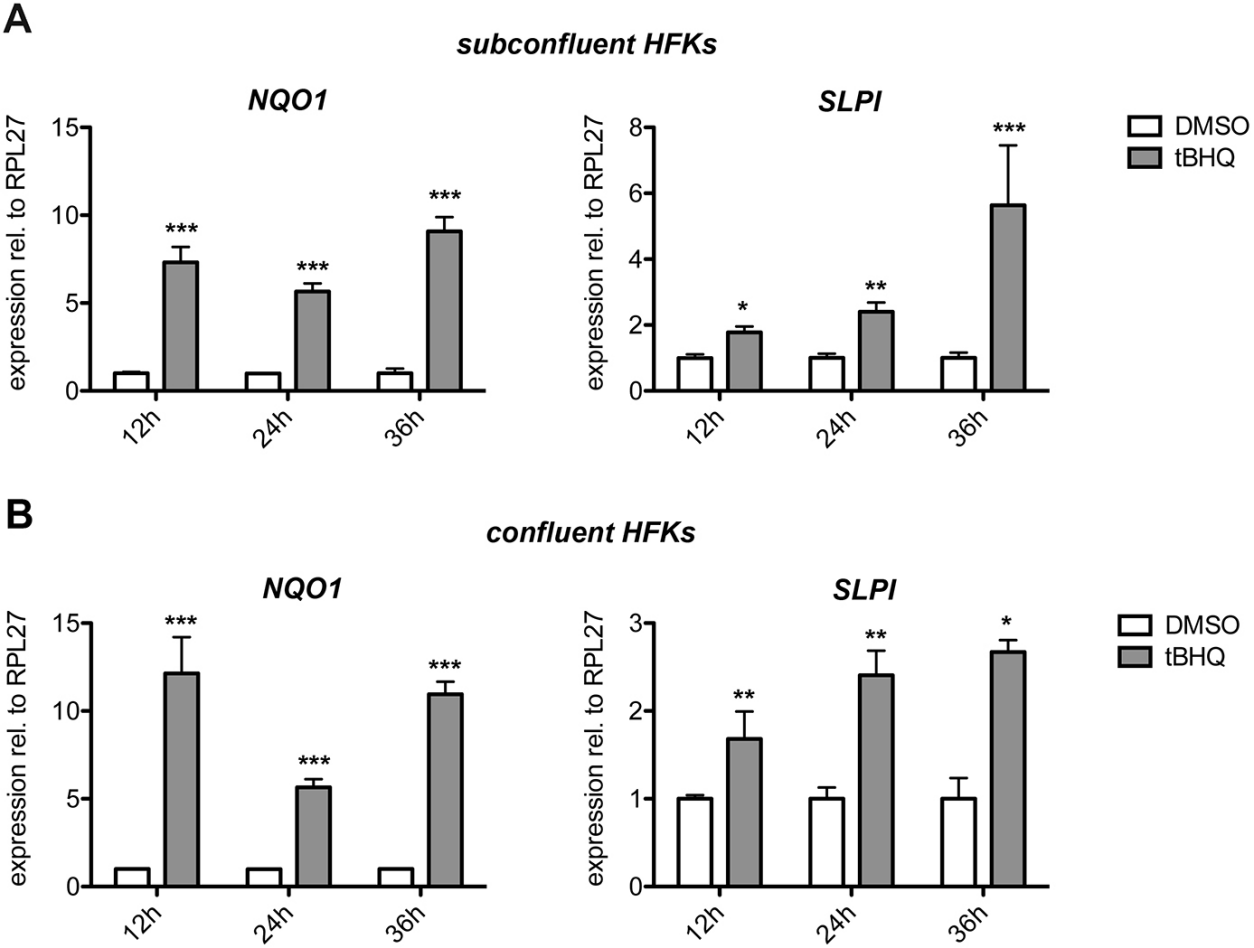
**Pharmacological NRF2 activation induces expression of *NQO1* and *SLPI* in primary human keratinocytes.** (A,B) Human primary foreskin keratinocytes (HFKs) were grown to subconfluency (A) or confluency (B) and treated for 12 h, 24 h or 36 h with *t*BHQ (50 µM) or vehicle (DMSO). Cells were harvested and analyzed by qRT-PCR for *NQO1* (positive control), *SLPI* or *RPL27* (encoding ribosomal protein L27; used for normalization). *n*=3 per time point and treatment group; the result was reproduced in an independent experiment. All bars represent mean±s.d. *P≤0.05, **P≤0.01, ***P≤0.001, Student's *t*-test.

In summary, the loss of a functional *Spink5* gene and subsequent loss of LEKTI drives the uninhibited activation of the proteases Klk5, Klk7, Klk14 and Ela2 in the epidermis. Our experiments showed that genetic Nrf2 activation in the keratinocytes of Spink5ko mice partially restored epidermal integrity, probably due to the strong upregulation of Slpi (an inhibitor of Klk7 and Ela2). The resulting stabilization of (corneo)desmosomes promotes the attachment of the *stratum corneum*. Thus, the epidermal barrier function is restored and the upregulation of pro-inflammatory cytokines and chemokines is reduced (working model in Fig. 4E). Importantly, the NRF2-activating compound *tert*-butylhydrochinone (*t*BHQ) also increased expression of *SLPI* in subconfluent and confluent primary human foreskin keratinocytes (Fig. 5A,B), although the induction was delayed compared with induction of the classical NRF2 target gene *NQO1* (Fig. 5A,B). Together, the findings obtained with our mouse model and with cultured human keratinocytes suggest the possible use of pharmacological NRF2 activators for the treatment of Netherton syndrome.

## DISCUSSION

Netherton syndrome is a life-threatening disorder for which no efficient targeted therapies are available. We show here that activation of Nrf2 in the keratinocytes of Spink5ko mice strongly improves their cutaneous phenotype, primarily by ameliorating the epidermal barrier defect.

Several differences were observed between the phenotype of the Spink5ko mice used in this study (Yang et al., 2004) and other Netherton syndrome mouse models (Descargues et al., 2005; Furio et al., 2015), including a more generalized detachment of the *stratum corneum* concomitant with a normal expression of some pro-inflammatory markers, such as *Il1b*, and the lack of an inflammatory cell infiltrate. Although the primary clinical features were comparable across all published models, the observed differences could be attributable to potential effects of the insertion of a transposon in the model that we used, which led to the deletion of a 66.8 kb region of genomic DNA (Yang et al., 2004). Alternatively, the different genetic background, FVB/N (Yang et al., 2004), C57BL/6 (Descargues et al., 2005; Hewett et al., 2005) or a mixed background (this study), and/or variations in the microbiome of the animal facilities might be responsible. The latter factor strongly affected the phenotype of other mutant mice with barrier function defects (Archer et al., 2019; Williams et al., 2019).

The most likely mechanism underlying the beneficial effects of Nrf2 activation is the Nrf2-mediated upregulation of Slpi, a potent inhibitor of the proteases Klk7 and Ela2 (Franzke et al., 1996). Klk7 has been implicated in the cleavage of corneodesmosomal proteins, such as Cdsn and Dsc1, during terminal differentiation of the epidermis (Caubet et al., 2004), and of Dsg1 in tumor cells (Ramani et al., 2008). Ela2 is a protease important for maintaining epidermal homeostasis and is responsible for degradation of the structural protein (pro-)filaggrin (Bonnart et al., 2010). Interestingly, transgenic mice overexpressing Ela2 also exhibit a barrier defect, which probably results from excessive (pro-)filaggrin processing combined with alterations in lipid metabolism; similar changes in lipid metabolism are seen in the skin of Netherton syndrome patients (Bonnart et al., 2010; Fartasch et al., 1999; van Smeden et al., 2014). Thus, the Nrf2-mediated upregulation of Slpi, and subsequent inhibition of Klk7 and Ela2, probably result in the stabilization of Cdsn and consequently of desmosomes and corneodesmosomes in differentiated cell layers. This hypothesis is supported by the broad Cdsn staining observed in the epidermis of Spink5ko/K5cre-CMVcaNrf2 mice, which extended to the *stratum corneum*. This finding is not a consequence of transcriptional activation of *Cdsn* by Nrf2, as *Cdsn* mRNA levels were not upregulated in mice expressing the *caNrf2* transgene (data not shown). Building on previous work showing the clinical potential of targeting Klk5 in Netherton syndrome (Furio et al., 2014, 2015), our work establishes KLK7 and ELA2 as promising targets for the treatment of Netherton syndrome patients through the activation of NRF2 and subsequent upregulation of SLPI. These findings corroborate results obtained by other groups with respect to the importance of Klk7 in the pathogenesis of Netherton syndrome (Kasparek et al., 2017).

However, Slpi overactivation and the subsequent inhibition of Klk7 and Ela2 is unlikely to be the only reason for the phenotype amelioration in Spink5ko mice, as Nrf2 activates several genes associated with barrier function. For example, expression of small proline-rich protein 2d, a component of the cornified envelope, is upregulated by Nrf2 and this normalized the *in utero* barrier defect in loricrin-deficient mice (Huebner et al., 2012; Koch et al., 2000). Moreover, Nrf2-mediated reduction of oxidative stress might contribute to the beneficial effect of Nrf2 activation, as seen previously in a mouse model for palmoplantar keratoderma, a characteristic feature of pachyonychia congenita, which results from mutations in the *K6*, *K16* or *K17* genes (Kerns et al., 2016). In future studies it will be important to determine the extent of Nrf2 activation that is required to activate the different protective effects in this model.

The Nrf2-mediated restoration of the epidermal barrier function in Spink5ko mice was remarkable, but not complete. This observation could be due to incomplete Cre-mediated recombination in neonates, resulting in variable expression levels of caNrf2 across the epidermis, as shown earlier for the K5cre-CMVcaNrf2 mouse line (Schäfer et al., 2014).

The selective Toluidine Blue uptake in the extremities of Spink5ko/K5cre-CMVcaNrf2 mice is intriguing, however. As mouse fetuses move their extremities, including their heads and tails (Kahn et al., 2009; Suzue, 1996), these areas might be subject to greater mechanical stress *in utero*, thereby precluding an efficient attachment of the cornified layers. Nonetheless, even a partial restoration of the epidermal permeability barrier function reduced the expression of crucial pro-inflammatory cytokines. The reduction of *Tslp* and *Il6* expression is probably a consequence of the suppression of TNF-α expression/signaling (Bogiatzi et al., 2007; He and Geha, 2010). Interestingly, neutralizing TNF-α antibodies (Infliximab) have shown promising results in clinical studies and reduced TSLP expression in patients (Fontao et al., 2011; Roda et al., 2017). TNF-α, as well as Par2, activate the NFκB transcription factor (Briot et al., 2009, 2010); the overactivation of Klk5 in the epidermis of Spink5ko mice also activated this pathway, resulting in the upregulation of *Icam1* and *Tslp* (Briot et al., 2009). This finding is in agreement with the increased expression of these genes observed in Spink5ko mice, and suppression to basal levels upon Nrf2 activation.

Although only the effects of a keratinocyte-specific activation of Nrf2 were investigated in this study, Netherton syndrome is a multi-organ disorder with a strong immune component (Hintner et al., 1980; Renner et al., 2009). Patients often present with hyper-IgE syndrome (Saenz et al., 2018; Smith et al., 1995; Wilkinson et al., 1964), eosinophilia (Hovnanian, 2013; Sun and Linden, 2006; Williams et al., 2015), defects in adaptive immunity (Renner et al., 2009; Stryk et al., 1999) and natural killer cell toxicity (Renner et al., 2009). This immune system dysfunction could underlie the inability of Nrf2 activation to rescue the upregulation of pro-inflammatory molecules, such as S100a8 and S100a9, in Spink5ko mice, although this might also result from persistent epidermal dehydration in Spink5ko/K5cre-CMVcaNrf2 pups (Zhong et al., 2016). Likewise, we did not observe a change in the number of immune cells infiltrating the skin; however, this again could be attributed to the analysis of the mice shortly after birth, which precludes the development of a full-blown immune response.

The effect of genetic Nrf2 activation on the survival of Spink5ko mice was not investigated, as it was precluded by local animal welfare regulations. Our study nonetheless shows a remarkably positive effect of activated Nrf2, which could be clinically relevant. Although it needs to be taken into consideration that long-term continuous activation of Nrf2, as seen in our caNrf2 transgenic animals, caused defects in the cornified envelope, mild inflammation and cyst formation (Schäfer et al., 2012, 2014); this might not be the case in mice with an impaired epidermal barrier, as suggested by the rescue of the barrier function deficiency in loricrin knockout mice by pharmacological Nrf2 activation (Huebner et al., 2012). In a treatment setting, potential adverse effects of activated NRF2 on the epidermis could be controlled by the frequency and dose of NRF2-activating compounds.

Multiple novel targeted treatment strategies for Netherton syndrome are currently in development or under investigation, and some strategies are based on correcting the genetic defects implicated in Netherton syndrome by using gene therapy (Di et al., 2011, 2013, 2019; Roedl et al., 2011). Although such therapies offer the possibility of a cure, they are likely to face many regulatory hurdles on the path to the clinic. By contrast, NRF2 activators are easy to combine with any of the above-described therapy regimens and could work orthogonally to previously established modes of treatment, potentially resulting in an additive effect. Importantly, the NRF2-activating compound sulforaphane induced *SLPI* expression in human nasal epithelial cells in an NRF2-dependent manner (Meyer et al., 2013), and *t*BHQ treatment of primary human keratinocytes also promoted *SLPI* expression (this study). Although an NRF2-independent upregulation of *SLPI* by *t*BHQ in keratinocytes cannot be excluded, the strong effect of *t*BHQ on the expression of the classical NRF2 target gene *NQO1* observed in the same experiment, and the presence of several NRF2-binding sites in the human *SLPI* promoter, strongly suggest that the effect is mediated by NRF2. Unfortunately, a potential effect of Nrf2-activating compounds could not be tested in the neonate mice, because of the short observation period. Such compounds might well be successful in Netherton syndrome patients, however, who survive despite their severe symptoms. Thus, topical treatment of these patients with pharmaceutical preparations containing synthetic or natural NRF2 activators (Reisman et al., 2015; Rojo de la Vega et al., 2018) provides a promising therapeutic concept.

## MATERIALS AND METHODS

### Animal experiments

Mice were housed under specific pathogen-free conditions and received water and food *ad libitum*. Care and use of experimental animals complied with Swiss animal welfare law. Mouse experiments and maintenance of transgenic mouse lines were approved by the local veterinary authorities (Kantonales Veterinäramt Zürich).

Spink5ko(he) mice (FVB/N) were crossed with K5cre mice (C57BL/6) and in parallel with CMV-caNrf2 mice (FVB/N). In the next step, Spink5ko(he)/K5Cre mice (FVB/N×C57BL/6 F1), which had been further backcrossed for one generation with K5Cre mice, were crossed with Spink5ko(he)/CMvcaNrf2 (FVB/N) mice. Neonatal mice were sacrificed by decapitation or pentobarbital injection, and isolated total skin or epidermis were used for further analysis. Animals were genotyped using DNA extracted from tail biopsies. DNA was amplified by PCR using the KAPA2G FAST Genotyping Mix (Kapa Biosystems, Wilmington, MA, USA) and the amplified fragments were visualized by gel electrophoresis. Primers used for PCR amplification are listed in Table S1.

### Dermis-epidermis separation from neonatal mouse skin

Separation of epidermis from the dermis was achieved by heat shock treatment of mouse back skin [30 s at 60°C followed by 1 min at 4°C, both in phosphate-buffered saline (PBS)]. The epidermis was gently peeled off using forceps and homogenized for RNA isolation, as described below.

### RNA isolation and qRT-PCR analysis

Isolation of mRNA was performed according to the manufacturer's instructions (MinElute kit, Qiagen, Hilden, Germany). cDNA synthesis and qRT-PCR analysis were performed exactly as previously described (Schäfer et al., 2012). Primer sequences are provided in Table S1.

### Histological and immunofluorescence staining

Hematoxylin and Eosin (H&E), Toluidine Blue and immunofluorescence staining were performed as previously described using 7 µm paraffin sections of skin fixed in 4% paraformaldehyde or acetic ethanol (95% ethanol/1% acetic acid) (Antsiferova et al., 2013; Schäfer et al., 2010). The primary and secondary antibodies used for immunostaining are listed in Table S2. Image acquisition was performed using either a Zeiss Axioskop 2 microscope/Axiocam HRc camera (bright-field) or a Zeiss AxioImager.A1 microscope/Axiocam MRm camera (fluorescence images); Axiovision software was employed in both cases (Zeiss, Oberkochen, Germany). Analysis was performed blinded by the investigator.

### Epidermal barrier function assays

For outside-in barrier function analysis, euthanized neonates were dehydrated by sequential bathing in 25%, 50% and 75% methanol in PBS, followed by pure methanol for about 1 min to extract polar lipids. The pups were then rehydrated by bathing in reverse order, followed by incubation in PBS. Staining was carried out overnight in Toluidine Blue O (0.1% solution in PBS) followed by destaining in PBS; the pups were then photographed (Furio et al., 2015; Hardman et al., 1998; Schäfer et al., 2012).

Analysis of inside-out barrier function was carried out by measuring TEWL using a Tewameter^®^ 3000 (Courage and Khazaka, Cologne, Gemany). The average of 20-30 readings per pup was calculated after the measurement reached a plateau (Schäfer et al., 2012).

### Ultrastructural analysis

An EM109 electron microscope (Zeiss, Oberkochen, Germany) was used to acquire ultramicroscopy micrographs. Sample preparation was performed as described earlier (Yang et al., 2010).

### Culture and treatment of primary human keratinocytes

Human foreskin keratinocytes from healthy boys without signs of skin disease were established and cultured as previously described (Sollberger et al., 2012). Cells between passage 3 and 5 were seeded in keratinocyte serum-free medium (Gibco BRL, Paisley, UK), supplemented with epidermal growth factor and bovine pituitary extract (Gibco BRL). Cells were grown to subconfluency or confluency in six-well culture dishes and treated for 12 h, 24 h or 36 h with the NRF2-activating compound *t*BHQ (50 µM; Sigma, Munich, Germany) or vehicle (DMSO). Treated cells were analyzed by qRT-PCR for expression of *SLPI* and *NQO1* relative to *RPL27*. The confluency of the cells was important, as *in vitro* differentiated human keratinocytes expressed very high levels of *SLPI*, which did not allow a further increase by NRF2 activation (data not shown).

### Statistical analysis

Statistical analyses were performed using the non-parametric Mann–Whitney *U*-test for non-Gaussian distribution using Prism (Version 5, GraphPad Software, La Jolla, CA, USA), as the residuals of most data sets were not normally distributed precluding the use of two-way ANOVA. All data points were included in the analysis. Error bars represent s.d. *P≤0.05, **P≤0.01, ***P≤0.001.
